# A Descriptive Study of Repeated Hospitalizations and Survival of Patients with Metastatic Melanoma in the Northern Italian Region during 2004–2019

**DOI:** 10.3390/curroncol30060400

**Published:** 2023-05-25

**Authors:** Matilde Mannucci, Vincenzo Fontana, Dalila Campanella, Rosa Angela Filiberti, Paolo Pronzato, Alessandra Rosa

**Affiliations:** 1 Clinical Epidemiology Unit, IRCCS Ospedale Policlinico San Martino, 16132 Genoa, Italy; 2 Medical Oncology Unit, IRCCS Ospedale Policlinico San Martino, 16132 Genoa, Italy

**Keywords:** melanoma, metastasis, hospitalization, rehospitalization, healthcare, survival

## Abstract

Background: Survival rates for metastatic melanoma (MM) patients have improved in recent years, leading to major expenses and health resource use. We conducted a non-concurrent prospective study to describe the burden of hospitalization in a real-world setting for patients with MM. Methods: Patients were tracked throughout all hospital stays in 2004–2019 by means of hospital discharges. The number of hospitalizations, the rehospitalization rate, the average time spent in the hospital and the time span between consecutive admissions were evaluated. Relative survival was also calculated. Results: Overall, 1570 patients were identified at the first stay (56.5% in 2004–2011 and 43.7% in 2012–2019). A total of 8583 admissions were retrieved. The overall rehospitalization rate was 1.78 per patient/year (95%CI = 1.68–1.89); it increased significantly with the period of first stay (1.51, 95%CI = 1.40–1.64 in 2004–2011 and 2.11, 95%CI = 1.94–2.29 thereafter). The median time span between hospitalizations was lower for patients hospitalized after 2011 (16 vs. 26 months). An improvement in survival for males was highlighted. Conclusions: The hospitalization rate of patients with MM was higher in the last years of the study. Compared with a shorter length of stay, patients were admitted to hospitals with a higher frequency. Knowledge of the burden of MM is essential for planning the allocation of healthcare resources.

## 1. Introduction

Malignant melanoma is the most aggressive form of cancer, responsible for 80% of deaths from skin neoplasms [1]. The incidence of all-stage melanoma has increased globally [2]. In Italy, these tumors account for approximately 4% of all incident tumors, with a higher frequency, up to 9%, in people younger than 50 years. In the northern regions, incidence (age-standardized to the European population) is roughly 20 per 100,000 person-years [3]. Incidence showed a constant increase from 1994 to 2013, with annual percent changes of 3.6 and 2.5 estimated among men and women, respectively; however, a stabilization or a decrease under the age of 35, possibly due to sun-protective behaviors at younger ages, has occurred [4].

The natural history of melanoma is heterogeneous, with differences in disease progression and sites of metastatic lesions, and a multimodal therapeutic approach is often required [5]. Tumor cells disseminate easily beyond the primary site, and advanced-stage, unresectable or metastatic tumors have a poor prognosis. Melanoma is a highly immunogenic tumor, and the introduction of immunosuppressive drugs along with the availability of various effective treatments such as ipilimumab and checkpoint inhibitors has brought substantial clinical efficacy, showing encouraging results in the life expectancy of most metastatic melanoma (MM) patients [5,6,7]. Notably, the likelihood of receiving immunotherapy has increased in younger patients with low comorbidities treated at research medical centers [8].

The increased number of people living after a diagnosis of MM has an impact on healthcare facilities and is associated with major economic expenses and health resource use [1,9,10,11]. Investigating the frequency of hospitalizations may be useful to understand the performance of the healthcare system, take actions for healthcare planning and then improve the effectiveness of the everyday clinical management of MM patients.

We conducted a non-concurrent prospective study to quantify and describe the burden of hospitalization in a real-world setting in patients diagnosed with MM in an Italian region, comparing two periods, before and after 2012, which can be approximately assumed as the moment in which the abovementioned treatments were introduced.

The first objective was to explore the timing of hospitalization along with the main reasons (melanoma or other conditions), frequency and patterns of readmission of MM patients. The second objective was to evaluate survival among non-selected patients with this tumor in order to estimate improvements in life expectancy attributable, to some extent, to the new therapeutic options.

## 2. Material and Methods

### 2.1. Study Patients

The sources used in this study were the Hospital Discharge Records (HDRs) of the Liguria region (LR), which contain information on all patients discharged from hospitals after a planned or urgent (diagnostic or interventional) admission. In Italy, healthcare is covered almost entirely by the National Health Service (NHS), and the use of this database makes it possible to track virtually all the hospitalizations in the country. Although HDR were primarily intended for administrative purposes to obtain refunds, now they are also used for epidemiological, public health and clinical purposes [12,13]. LR is one of the 20 administrative regions of Italy, located in the north-west, with a population of approximately 1.6 million inhabitants.

The database records demographic characteristics, hospital and department admission codes, type of hospitalization (ordinary or day hospital), admission and discharge dates, clinical procedures and discharge status (in-hospital death or recovery). The primary diagnosis at discharge and up to seven secondary comorbidities are reported. These diagnoses are codified at each hospital according to the Italian edition of the International Classification of Diseases, 9th Revision, Clinical Modification (ICD-9-CM).

The patients included in this study were discharged from hospital between 1 January 2004 and 31 December 2019 with a diagnosis of MM. We searched for patients starting in 2004 due to the low accuracy and comparability of HDR diagnostic codes prior to that.

To identify MM, we searched for ICD-9-CM codes 172 (malignant melanoma of skin) or v10.82 (medical history of melanoma) associated with codes 196 to 199 (secondary malignant neoplasm), located in any position of discharge diagnosis. Codes v58.1 (chemotherapy), 99.25 (injection or infusion of cancer chemotherapeutic substance) and 99.28 (immunotherapy agent injection) were also extracted (Table 1).

The first admission with discharge diagnosis of melanoma of skin associated or not with secondary malignant neoplasm in the period of interest was used as an index of first hospitalization (H_0_) for MM. Each Italian resident has a unique identification code (fiscal code), which was used to track all individual hospital stays independently from the ICD code. In this way, stays that were not considered directly related to MM were also retained.

The discharge data on patients resident in LR but hospitalized in other Italian regions were also collected. Patients were never directly involved.

Patients were followed up until the end of 2019. Vital status was documented through the LR Mortality Registry, active since 1988, municipal demographic databases and HDR discharge status.

This study was approved by the Ethics Committee of LR (study 537/2021, ID 11809, Ospedale Policlinico San Martino) and was conducted in compliance with the principles of the Declaration of Helsinki. HDR are recorded with the patient’s informed consent and can be used as aggregated data for scientific studies without further authorization.

### 2.2. Study Aims

The primary endpoint of the study was to characterize hospital admissions during the course of the disease in patients with MM. The main cause of readmission (melanoma or other conditions), total number of hospitalizations and hospitalization rates by gender, age at H_0_ and period of H_0_ were evaluated together with the number of centers at H_0_ and subsequent admissions. In order to characterize the longitudinal trend of hospitalization, the average time spent in the hospital and time span between consecutive admissions were calculated by gender, age and period.

As a secondary endpoint, survival probabilities were also calculated to assess changes in time trends of life expectancy allegedly attributable at least partially to changes in the therapeutic regimens of MM patients.

### 2.3. Statistical Analysis

HDR data were analyzed according to patients’ baseline main characteristics, namely gender, age at H_0_, period of H_0_ (2012–2019 vs. 2004–2011) and vital status at the end of follow-up. Age was split into four categories according to the quartiles (52, 64 and 74 years) of the frequency distribution.

The median time between two consecutive admission dates, along with the interquartile range (IQR), was used as an index of healthcare burden attributable to multiple admissions of MM patients. In order to reduce random variability in median estimates due to a small sample size, only patients with 15 or fewer readmissions were considered for analyses. This selection amounted to excluding less than 4% (261 out of 7013) of all hospitalizations.

Additionally, a multivariable negative-binomial regression analysis was applied to rehospitalization rates in order to evaluate the joint effect of patients’ baseline characteristics while adjusting regression estimates for confounding and allowing for overdispersion in count data [14]. The likelihood ratio test was applied to assess the statistical significance (*p*-value < 0.05) of all regression estimates.

In addition, the 2-year and 5-year life expectancies were evaluated by gender, age and period using relative survival (RS) rates estimated through the Ederer II method [15]. RS is derived as a ratio between observed (OS) and expected (ES) survival probabilities: the former estimated using the life table method and the latter based on the annual general mortality rates of LR in 2004–2019 stratified by age and gender, downloaded from the Italian Statistical Institute (ISTAT) website (http://demo.istat.it/ (accessed on 1 April 2021)). It is worth noting that RS represents a correct estimate of net survival measure in the presence of a sizeable competing risk of dying from causes other than MM and in the absence of an accurate cause-of-death certification [15].

All statistical estimates were accompanied by 95% confidence intervals (95%CI) as a measure of sampling variation. Data analysis was carried out using Stata software (StataCorp. Stata: Release 17. Statistical Software. College Station, TX, USA, 2021).

## 3. Results

Overall, 1570 patients with MM (57.2% males) were identified at first stay (56.5% in the years 2004–2011 and 43.7% in the years 2012–2019). Approximately 27% of admissions were related to patients younger than 53 years, while approximately 29% concerned patients aged 74 years or older (Table 2).

A total of 8583 admissions were retrieved, along with a median of 3 (IQR = 1–6) hospitalizations per patient: 1570 (18%) were first admissions and 7013 (82%) were readmissions. Approximately 90% of patients were rehospitalized at least once, and roughly 30% of them had up to 6 stays. Only ca. 2% experienced 15 hospitalizations.

Generally, the percentage of rehospitalizations was lower for patients diagnosed during 2012–2019 as compared to 2004–2011 (88% vs. 93% at the second admission, 72% vs. 83% at the fifth and 66% vs. 72% at the tenth).

Overall, the patients were treated in 162 Italian hospitals (29 in LR, with hospitalization for 90% of the patients): 41% in one (30% in the first period and 56% in the second), 32% in two, 18% in three and 9% in four or more hospitals (14% in the first period and 3% in the second). The percentage of patients hospitalized outside the LR was approximately 10% in 2004–2011 and 9% afterwards. The main cause of total hospitalization was MM (75% at the second stay, roughly 60% for almost all admissions and up to 76% in the last admission) (Table S1: Cause of hospitalization for patients with MM in LR during 2004–2019).

Given the large number of other causes of admission, they were put together in one category. In the whole sample, the median length of hospital stay was 29 days; it was longer for patients of intermediate ages (approximately 35 days) and for those hospitalized in the first period (32 vs. 24 days) (Table 3).

**Figure 1:**
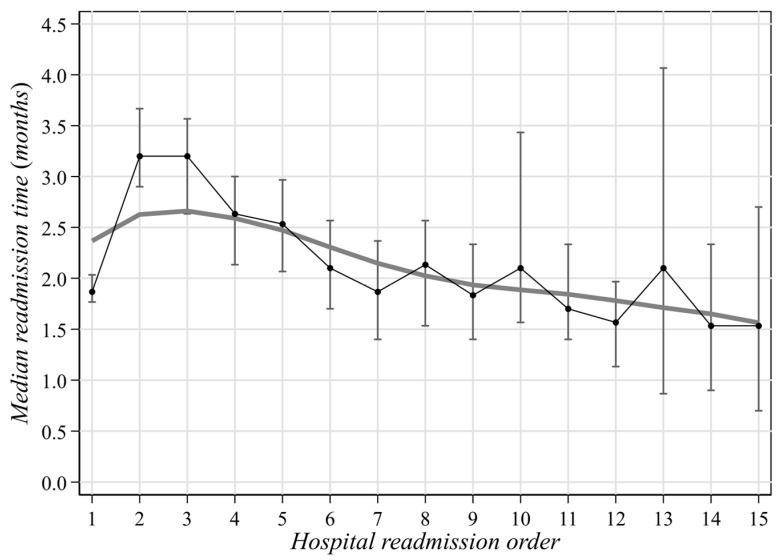
Overall median time span (with 95%CI) between two consecutive hospitalizations of MM patients in Liguria Region during 2004–2019.

**Figure 2:**
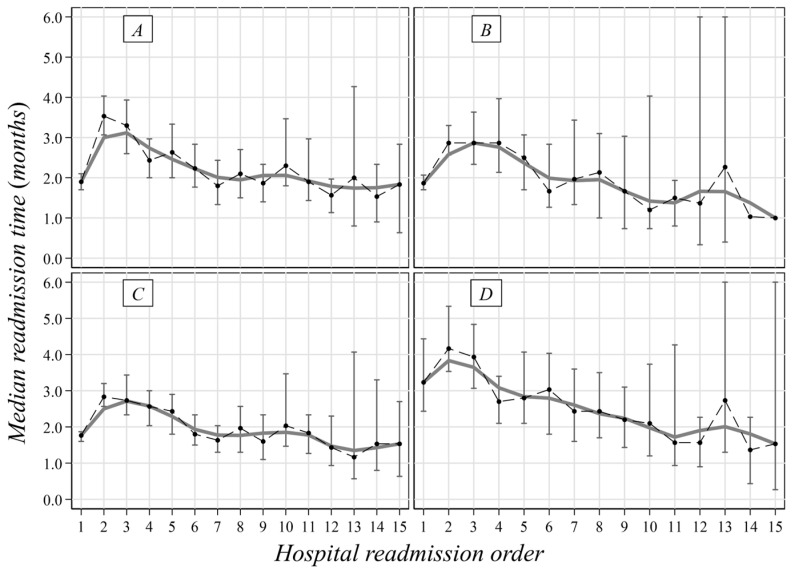
Median time span (with 95%CI) between two consecutive hospitalizations of MM patients in Liguria Region during 2004–2019 by period and cause. (**A**) First admission in 2004–2011; (**B**) first admission in 2012–2019; (**C**) melanoma as a cause of admission; and (**D**) other causes of admission.

The median time span between hospitalizations was 20 months, and the period shortened along with the number of hospitalizations (Figure 1). No remarkable median differences by gender were pointed out (males: 20, IQR: 6–45 vs. females: 22, IQR = 7–54), while the median period was shorter in older people (Table 3). A reduced time span (roughly 2 months) was also observed for patients admitted because of MM (Figure 2) and among patients hospitalized after 2011 (16 months vs. 26 months in the previous 8 years) (Table 3, Figure 2).

A total of 23% of first admissions were in medical wards, and 61% were in surgery. In total, 13% of patients were hospitalized in an oncology ward, and the percentage increased to more than 35% in the following admissions (Table S2: Wards of hospitalization for patients with MM in Liguria Region during 2004–2019).

Data on the type of hospitalization were scarce, being reported in approximately 50% of HDR. According to these data, more than 40% of hospitalizations at first admission had been planned, but this percentage decreased after two hospitalizations, together with an increase in admissions due to emergency situations (from 11% to approximately 30% at the tenth stay) (Table S3: Type of hospitalization for patients with MM in Liguria Region during 2004–2019).

In all stays, on average, more than 50% of patients were hospitalized on an ordinary regimen, and the remaining attended a day hospital. Mainly, patients were admitted to day hospitals to undergo minor surgery (48%) or therapy (28%); the percentage of surgery decreased with subsequent hospitalizations, together with a rise in patients undergoing therapies (roughly 70%). This increase was particularly evident in 2012–2019 (Table S4: Reasons for day-hospital admissions for patients with MM in Liguria Region during 2004–2019). Overall, 12% of patients underwent chemotherapy during their first stay and up to 30% during the following admissions. The percentage of these patients was somewhat higher in 2012–2019 (approximately 40%) compared to 2004–2011 (approximately 30%) (Table S5: Chemotherapy in patients with MM in Liguria Region during 2004–2019).

**Figure 3:**
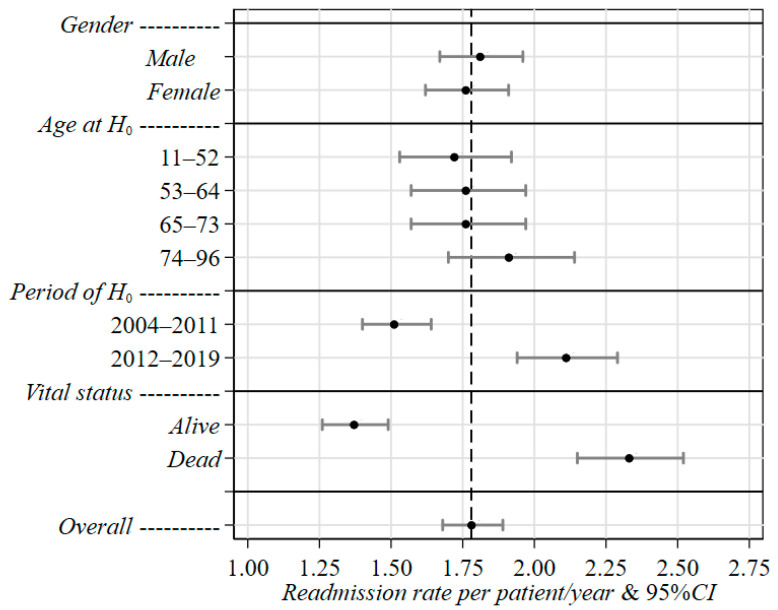
Joint effect of gender, age at H_0_, period of H_0_ and vital status at last discharge on readmission rates of MM patients in Liguria Region during 2004–2019, estimated through the multivariable negative-binomial regression method. 95%CI: 95% confidence intervals for rate. Vital status: vital status at last discharge.

The negative-binomial regression estimates are reported in Table 4 (Figure 3) as readmission rates and rate ratios (RR), along with the corresponding 95%CIs and *p*-values. Such a statistical approach allowed us to estimate an overall rate of 1.78 per patient/year (95%CI = 1.68–1.89). Differences in the risk of new admission by gender and age at H_0_ were found to be rather small. In particular, females experienced a rate (1.76, 95%CI = 1.62–1.91) slightly lower than males (1.81, 95%CI = 1.67–1.96), while a remarkable increase with age (+11%) was found only for older patients (74–96 years: 1.91, 95%CI = 1.70–2.14) when compared to younger patients (11–52 years: 1.72, 95%CI = 1.53–1.92). A total of 56% of patients had died by the end of the study period, and, as expected, such subjects showed a rate that was roughly 70% higher (2.33, 95%CI = 2.15–2.51) than that (1.37, 95%CI = 1.26–1.49) estimated for patients who were still alive.

As far as the period of H_0_ is concerned, a noteworthy difference in readmission rates was pointed out. Specifically, patients with a first hospitalization in 2012–2019 showed a readmission rate of 2.11 (95%CI = 1.94–2.29), which was 40% higher than that estimated in 2004–2011 (1.51, 95%CI = 1.40–1.64) (Table 4, Figure 3).

**Figure 4:**
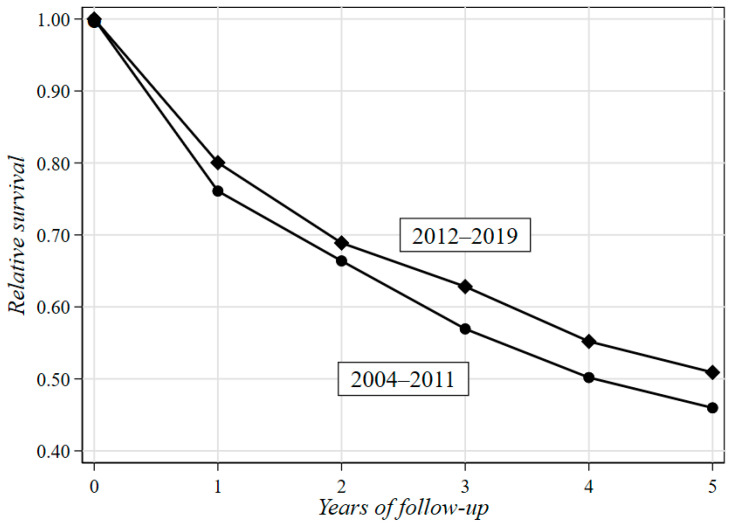
Difference in relative survival rates between the two periods of H_0_ (2012–2019 vs. 2004–2011) over a five-year follow-up.

Table 5 shows the results of the RS analysis stratified by gender, age at H_0_ and period of H_0_. In order to obtain OS estimates based on comparable follow-up periods, patients with H_0_ in 2004–2011 were followed up until 31 December 2011. As a consequence, patients who did not die by the end of 2011 were considered censored, regardless of the actual outcome they experienced afterwards. Figure 4 illustrates the difference in RS between the two periods of H_0_ (2012–2019 vs. 2004–2011) over a five-year follow-up. The two-year RS rate of the whole cohort of patients was 67% (95%CI = 64–70%). A clear tendency towards an increase was observed in both sexes in patients under 52 years from 2012 to 2019 (69% vs. 82% for males and 70% vs. 82% for females). The five-year RS was 48% (95%CI = 45–52%), and a tendency towards an increase was observed for males in all age groups, mostly in the range of 65–73 years (41% vs. 62%), between 2012 and 2019. By contrast, only younger females (11–52 years) showed a positive trend in RS rates (53% vs. 59%), while in the other age groups, rates were either invariant (65–73 years) or decreasing (53–64 and 74–96 years).

## 4. Discussion

This is a preliminary descriptive study designed to explore the burden of hospitalizations in patients with a diagnosis of MM in a northern Italian region. To achieve this aim, we performed an analysis of the discharges of patients hospitalized in the periods 2004–2011 and 2012–2019 in private or public health centers because of a tumor or other causes. Patients’ demographics as well as hospitalization characteristics were evaluated.

According to our results, more than 50% of patients were hospitalized in a high-volume hospital and were admitted in more than 60% of cases because of the tumor. The migration rate was approximately 10%. The number of admissions ranged from 1 to 67, with a median of 3, and 2% of subjects had at least 15 hospitalizations. The rehospitalization rate was higher for males and older people and increased remarkably with the period of H_0_. From 2012 onward, the length of stay was shorter with respect to the previous period (24 vs. 32 days), although a shorter median time span between hospitalizations (16 vs. 26 months) was highlighted. In addition, over the study period, we observed a decreasing trend in the number of hospitals in which patients were assisted. In particular, the frequency of patients who stayed in more than two hospitals decreased from 37% in 2004–2011 to 14% afterwards. The two- and five-year overall RS rates were 67% and 48%, respectively, and a more favorable prognosis was seen in younger and male patients diagnosed in 2012–2019 compared with those diagnosed in 2004–2011. However, such a tendency was not observed in females.

The estimated five-year net overall melanoma survival in Italy is approximately 87% [3], while in Western Europe, RS ranges from 85% to 90% (stage I: 95% to 100%; stage II: 65% to 90%) [16,17,18]. Nevertheless, disease recurrence develops in more than 10% of patients with localized melanoma and in more than 50% of those with regionally advanced melanoma. Approximately 20% of patients progress to metastatic disease [19,20,21,22]. Prognosis remains poor for these patients, with five-year OS rates ranging from 40% to 70% in stage III and from 9% to 28% in stage IV [17,23,24].

However, over the last decade, new treatment options have changed the scenario for patients with advanced disease thanks to therapeutic approaches aimed at obtaining a longer clinical response and more effective disease control [25].

A study on more than half a million cancer patients aged 15–74 years in 1985–2011 collected from the population-based Italian cancer registry network estimated the life expectancy of melanoma and the proportions of patients with similar death rates to the general population (cure fraction). Life expectancy is an indicator of the disease burden and provides “real-world” estimations for the actual impact of the tumor on the population of interest. The median life expectancy for skin melanoma has generally increased over the last 10 years by approximately 13% in men and 6% in women and was roughly 3 and 5 years, respectively, for cancer cases diagnosed in 2000. Furthermore, the cure fraction was greater than 75% in both sexes [26,27,28].

It has been estimated that in Italy, as of 2016, approximately 2000 patients had MM, and from 70% to 90% of them had been treated with innovative therapies, with approximately 30% entering compassionate use and expanded access programs [29].

Over the last 10 years, the estimated median survival time of patients with advanced stages has increased from approximately 6–9 to 18–23 months across all patients and up to 38 months in treatment-naive patients, as demonstrated in clinical trial data as well as in real-life practice. The estimated five-year survival rate ranges from 34% to 41%, and over 50% of patients reach a second line of treatment [25,30,31,32,33,34,35,36,37,38,39,40,41].

Our results were consistent with these data, even if the improvement in prognosis from 2012 onward was seen only in males and younger ages without having the possibility of distinguishing between regional lymph node involvement and more advanced disease. We assumed that all patients died from the cancer, and although the prognosis for MM patients is poor, an overestimation of mortality might have occurred.

Improvement in prognosis is also consistent with the greatest chance for young people to receive immunotherapy. In addition, our results showed an increase in admissions to oncologic wards and in chemotherapies from 2012 onward, together with a decreasing trend in the number of hospitals in which patients were assisted. This is suggestive of a centralization of healthcare with an improvement in patients’ outcomes.

Whilst new therapies have improved the survival of MM patients, they have also raised new challenges in terms of toxicity, being associated with immune-related adverse events requiring treatment [30,42,43]. In this regard, it has been observed that patients followed up for a minimum of 12 months from initiation of immunotherapy without disease progression had a low health-related quality of life and may face chronic conditions or exposure to high doses of steroids or other immunomodulatory drugs due to untoward medical events [43].

Generally, melanoma progression and changes in prognosis impact the use and allocation of healthcare resources and the economic burden [33,44].

To our knowledge, the surveys addressing overall hospitalization for MM in Italy are rare and rather outdated. According to the study by Maio et al. in 2012, only 10% of patients diagnosed in 2005–2006 with unresectable stage III or IV melanoma receiving systemic therapy and/or supportive care were hospitalized. Generally, hospitalization was more frequent for patients with any response to systemic therapy compared with those with no response (12% vs. 6%). On average, patients were hospitalized for approximately 30 days over a mean period of 17.5 months. Approximately 6% of patients received hospice care [1].

It has been observed that hospitalization costs related to melanoma increase approximately fourfold with disease progression; new therapies may delay the risk of relapse, but the acquisition cost and, in certain cases, their long-term use or drug toxicity increase the cost of melanoma care [9,42,44,45]. More recently, it has been observed that MM patients treated with at least one dose of the anti-CTLA-4 inhibitor ipilimumab need various types of healthcare services and that the economic burden associated with a drug-refractory disease is high. In Italy, 20% of patients had at least 1 hospitalization, and, apart from drug costs for systemic therapy, the total weekly costs for ipilimumab treatment after a mean follow-up of 37 weeks were EUR 91 [33].

A French study estimated that the annual per capita cost of hospitalization of MM patients in the post-progression stage increased up to threefold with respect to the pre-progression stage and that 27% of patients underwent hospitalization following adverse reactions to chemotherapy or immunotherapy [44]. Italian healthcare is covered almost entirely by the NHS, and on this basis, it is possible to reconstruct the hospitalization history of each resident. The use of HDR allowed for a view of the burden of MM on the hospitals of the entire LR, and it can be considered a systematic and cost-effective way of analyzing a large segment of the population.

Tracking hospitalization data is a tool for quantifying the impact on local hospital systems. Nevertheless, administrative healthcare databases can generate misclassifications in health services research and may not be accurate in giving information on patients’ health status. A diagnostic accuracy study showed that ICD-9-CM codes were able to correctly identify incident melanoma cases [12]. Nonetheless, the codes we used to find MM could not be so accurate for correctly identifying this pathology.

It is also possible that different ways of coding among physicians may lead to misclassification. This is one of the major problems with the statistical analysis of medical databases, and the only way to tackle the issue would be to make physicians aware of the epidemiological importance of strictly following the unified rules for disease coding.

Moreover, the rate of hospitalization due to MM may be overestimated since we searched for the codes used to identify MM among secondary diagnoses as well, and we arbitrarily considered all admissions with a discharge diagnosis not related to traumatisms as consequences of the study tumor. In so doing, we could have included some cases of other metastatic cancers in the analysis, and it is possible that not all the resources used during hospitalization were effective for the treatment of MM.

## 5. Conclusions

This study revealed that the hospitalization rate of patients with MM was higher from 2012 to 2019 with respect to the previous eight years. Compared with a shorter length of stay, patients were admitted to hospitals with a higher frequency. An improvement in survival rates was also highlighted, although it was limited to male patients. The introduction of new therapies has opened a new scenario for patients’ life expectancy, and knowledge of the burden of the disease is essential for planning the allocation of healthcare resources.

## Figures and Tables

**Table 1:** Selection of MM cases according to disease classification codes in discharge diagnosis.

ICD-9-CM Code ^a^	Description
172	Malignant melanoma of skin
V10.82	Medical history of melanoma
196–199	Secondary malignant neoplasm
V58.1	Chemotherapy
99.25	Injection or infusion of cancer chemotherapeutic substance
99.28	Immunotherapy agent injection

^a^ International classification of diseases, ninth revision, clinical modification.

**Table 2:** Characteristics of patients with MM at H_0_ in Liguria Region during 2004–2019.

Characteristics and Categories	Patients at H_0_^a^		Readmission			
	*N* ^b^	% ^c^	*N* ^b^	% ^c^	Median	IQR ^d^
Gender						
Male	898	57.2	3973	56.7	4	2–6
Female	672	42.8	3040	43.3	3	1–6
Age at H_0_ ^a^						
11–52	421	26.8	1870	26.7	3	2–6
53–64	343	21.8	1697	24.2	4	1–7
65–73	354	22.5	1795	25.6	4	2–7
74–96	452	28.8	1651	23.5	3	1–5
Period of H_0_ ^a^						
2004–2011	884	56.5	4617	65.8	4	2–7
2012–2019	686	43.7	2396	34.2	3	1–5
Vital status at last discharge						
Alive	689	43.9	2476	35.3	3	1–6
Dead	881	56.1	4537	64.7	4	2–7
Total	1570	100.0	7013	100.0	3	1–6

^a^ Index hospitalization (first admission); ^b^ absolute frequency; ^c^ percent frequency; ^d^ interquartile range.

**Table 3:** Hospitalization of patients with MM at H_0_ in Liguria Region during 2004–2019.

Characteristics and Categories	Days of Stay		Months between Hospitalizations	
	Median	IQR ^b^	Median	IQR ^b^
Gender				
Male	28	13–50	20	6–45
Female	30	13–52	22	7–54
Age at H_0_ ^a^				
11–52	25	8–43	22	6–54
53–64	33	13–58	21	7–56
65–73	36	20–59	27	9–55
74–96	25	13–44	14	5–36
Period of H_0_ ^a^				
2004–2011	32	16–56	26	9–64
2012–2019	24	8–45	16	4–38
Vital status at last discharge				
Alive	17	6–39	21	4–61
Dead	37	21–58	20	8–44
Total	29	13–51	20	7–49

^a^ Index hospitalization (first admission); ^b^ interquartile range.

**Table 4:** Joint effect of gender, age at H_0_, period of H_0_ and vital status at last discharge on readmission rates of MM patients in Liguria Region during 2004–2019, estimated through the multivariable negative-binomial regression method.

Characteristics and Categories	Rate ^c^	95%CI ^e^	RR ^d^	95%CI ^e^	*p*-Value ^f^
Constant ^a^	1.78	1.68–1.89	–	–	–
Gender					0.579
Male	1.81	1.67–1.96	1.00	(Ref.) ^g^	
Female	1.76	1.62–1.91	0.97	0.86–1.09	
Age at H_0_ ^b^					0.584
11–52	1.72	1.53–1.92	1.00	(Ref.) ^g^	
53–64	1.76	1.57–1.97	1.02	0.87–1.20	
65–73	1.76	1.57–1.97	1.03	0.87–1.20	
74–96	1.91	1.70–2.14	1.11	0.95–1.31	
Period of H_0_ ^b^					<0.001
2004–2011	1.51	1.40–1.64	1.00	(Ref.) ^g^	
2012–2019	2.11	1.94–2.29	1.40	1.24–1.57	
Vital status at last discharge					<0.001
Alive	1.37	1.26–1.49	1.00	(Ref.) ^g^	
Dead	2.33	2.15–2.51	1.70	1.52–1.91	

^a^ Constant: overall mean rate; ^b^ index hospitalization (first admission); ^c^ readmission rate per patient/year; ^d^ rate ratio; ^e^ 95% confidence interval for Rate/RR; ^f^ probability level of the likelihood-ratio test; ^g^ reference category.

**Table 5:** Life expectancy of MM patients in Liguria Region during 2004–2019.

Gender	Age at H_0_ ^a^	Period of H_0_ ^a^	Two-Year Life Expectancy				Five-Year Life Expectancy			
			OS ^b^	ES ^c^	RS ^d^	95%CI ^e^	OS ^b^	ES ^c^	RS ^d^	95%CI ^e^
Male	11–52	2004–2011	0.68	1.00	0.69	0.59–0.76	0.50	0.99	0.51	0.38–0.61
		2012–2019	0.81	1.00	0.82	0.71–0.89	0.57	0.99	0.58	0.44–0.68
	53–64	2004–2011	0.67	0.98	0.68	0.58–0.77	0.44	0.95	0.46	0.34–0.57
		2012–2019	0.73	0.99	0.74	0.60–0.84	0.50	0.96	0.52	0.36–0.66
	65–73	2004–2011	0.73	0.96	0.76	0.67–0.83	0.36	0.88	0.41	0.29–0.53
		2012–2019	0.74	0.96	0.77	0.65–0.86	0.56	0.90	0.62	0.47–0.74
	74–96	2004–2011	0.40	0.86	0.47	0.38–0.57	0.21	0.66	0.32	0.20–0.45
		2012–2019	0.40	0.87	0.46	0.34–0.57	0.25	0.68	0.38	0.23–0.52
Female	11–52	2004–2011	0.69	1.00	0.70	0.60–0.77	0.53	0.99	0.53	0.41–0.63
		2012–2019	0.82	1.00	0.82	0.71–0.90	0.58	0.99	0.59	0.43–0.70
	53–64	2004–2011	0.69	0.99	0.70	0.57–0.79	0.49	0.97	0.50	0.35–0.63
		2012–2019	0.68	0.99	0.69	0.54–0.79	0.45	0.98	0.45	0.28–0.61
	65–73	2004–2011	0.72	0.98	0.73	0.61–0.83	0.46	0.94	0.49	0.33–0.63
		2012–2019	0.61	0.98	0.63	0.47–0.75	0.46	0.94	0.49	0.32–0.64
	74–96	2004–2011	0.53	0.90	0.59	0.47–0.70	0.39	0.74	0.53	0.38–0.67
		2012–2019	0.56	0.91	0.62	0.49–0.73	0.37	0.78	0.47	0.31–0.62
Overall			0.64	0.96	0.67	0.64–0.70	0.43	0.89	0.48	0.45–0.52

^a^ Index hospitalization (first admission); ^b^ observed survival; ^c^ expected survival; ^d^ relative survival; ^e^ 95% confidence interval for RS.

## Data Availability

The datasets generated and analyzed during the current study are available from the corresponding author upon reasonable request. All statistical analyses were carried out using Stata software (StataCorp. Stata: Release 17. Statistical Software. College Station, TX. 2021).

## Supplementary Materials

The following supporting information can be downloaded at https://www.mdpi.com/article/10.3390/curroncol30060400/s1. Table S1: Cause of hospitalization for patients with MM in Liguria Region during 2004–2019. Table S2: Wards of hospitalization for patients with MM in Liguria Region during 2004–2019. Table S3: Type of hospitalization for patients with MM in Liguria Region during 2004–2019. Table S4: Reasons for day-hospital admissions for patients with MM in Liguria Region during 2004–2019. Table S5: Chemotherapy in patients with MM in Liguria Region during 2004–2019.
