# Supplementary material for: A Descriptive Study of Repeated Hospitalizations and Survival of Patients with Metastatic Melanoma in the Northern Italian Region during 2004–2019
Source: Curr Oncol. 2023 May 25;30(6):5266–78. doi: 10.3390/curroncol30060400 (PMC10297154; doi:10.3390/curroncol30060400)
Supplement: Supplementary file 1 [file curroncol-30-00400-s001.zip › Melanoma Current Onc Table S1.pdf]

Table S1. Cause of hospitalization for patients with MM in Liguria Region during 2004-2019.

Readmission	Period	MM		Other		Total
		N[b]	%[c]	N[b]	%[c]	
H_0[a]	2004-2011	884	100	0	0	884
	2012-2019	686	100	0	0	686
	Total	1570	100	0	0	1570
1	2004-2011	575	70	243	30	818
	2012-2019	487	81	116	19	603
	Total	1062	75	359	25	1421
2	2004-2011	466	66	241	34	707
	2012-2019	347	74	123	26	470
	Total	813	69	364	31	1177
3	2004-2011	406	66	209	34	615
	2012-2019	270	72	104	28	374
	Total	676	68	313	32	989
4	2004-2011	317	62	193	38	510
	2012-2019	184	68	86	32	270
	Total	501	64	279	36	780
5	2004-2011	259	63	152	37	411
	2012-2019	138	68	66	32	204
	Total	397	65	218	35	615
6	2004-2011	205	63	121	37	326
	2012-2019	102	69	46	31	148
	Total	307	65	167	35	474
7	2004-2011	144	58	106	42	250
	2012-2019	73	71	30	29	103
	Total	217	61	136	39	353
8	2004-2011	119	60	80	40	199
	2012-2019	52	71	21	29	73
	Total	171	63	101	37	272
9	2004-2011	87	61	56	39	143
	2012-2019	32	67	16	33	48
	Total	119	62	72	38	191
10	2004-2011	74	65	39	35	113
	2012-2019	21	70	9	30	30
	Total	95	66	48	34	143
11	2004-2011	53	59	37	41	90
	2012-2019	12	60	8	40	20
	Total	65	59	45	41	110
12	2004-2011	44	65	24	35	68
	2012-2019	6	43	8	57	14
	Total	50	61	32	39	82
13	2004-2011	30	58	22	42	52
	2012-2019	6	67	3	33	9
	Total	36	59	25	41	61
14	2004-2011	26	58	19	42	45
	2012-2019	4	67	2	33	6
	Total	30	59	21	41	51
15	2004-2011	24	83	5	17	29
	2012-2019	1	25	3	75	4
	Total	25	76	8	24	33

[a] First admission; [b] absolute frequency; [c] relative frequency (percentage).
