# Supplementary material for: A Descriptive Study of Repeated Hospitalizations and Survival of Patients with Metastatic Melanoma in the Northern Italian Region during 2004–2019
Source: Curr Oncol. 2023 May 25;30(6):5266–78. doi: 10.3390/curroncol30060400 (PMC10297154; doi:10.3390/curroncol30060400)
Supplement: Supplementary file 1 [file curroncol-30-00400-s001.zip › Melanoma Current Onc Table S2.pdf]

**Table S2.** Wards of hospitalization for patients with MM in Liguria Region during 2004-2019.

| Readmission | Period | Medical | | Surgery | | Oncology | | Other | | Missing | | Total |
|---|---|---|---|---|---|---|---|---|---|---|---|---|
| | | N[b] | %[c] | N[b] | %[c] | N[b] | %[c] | N[b] | %[c] | N[b] | %[c] | |
| | 2004-2011 | 234 | 26 | 503 | 57 | 127 | 14 | 19 | 2 | 1 | 0 | 884 |
| $H_0$[a] | 2012-2019 | 132 | 19 | 452 | 66 | 84 | 12 | 8 | 1 | 10 | 1 | 686 |
| | Total | 366 | 23 | 955 | 61 | 211 | 13 | 27 | 2 | 11 | 1 | 1570 |
| | 2004-2011 | 200 | 24 | 426 | 52 | 147 | 18 | 44 | 5 | 1 | 0 | 818 |
| 1 | 2012-2019 | 80 | 13 | 339 | 56 | 158 | 26 | 19 | 3 | 7 | 1 | 603 |
| | Total | 280 | 20 | 765 | 54 | 305 | 21 | 63 | 4 | 8 | 1 | 1421 |
| | 2004-2011 | 177 | 25 | 293 | 41 | 185 | 26 | 49 | 7 | 3 | 0 | 707 |
| 2 | 2012-2019 | 72 | 15 | 141 | 30 | 218 | 46 | 34 | 7 | 5 | 1 | 470 |
| | Total | 249 | 21 | 434 | 37 | 403 | 34 | 83 | 7 | 8 | 1 | 1177 |
| | 2004-2011 | 169 | 27 | 195 | 32 | 194 | 32 | 52 | 8 | 5 | 1 | 615 |
| 3 | 2012-2019 | 88 | 24 | 91 | 24 | 166 | 44 | 24 | 6 | 5 | 1 | 374 |
| | Total | 257 | 26 | 286 | 29 | 360 | 36 | 76 | 8 | 10 | 1 | 989 |
| | 2004-2011 | 149 | 29 | 140 | 27 | 179 | 35 | 40 | 8 | 2 | 0 | 510 |
| 4 | 2012-2019 | 70 | 26 | 68 | 25 | 115 | 43 | 15 | 6 | 2 | 1 | 270 |
| | Total | 219 | 28 | 208 | 27 | 294 | 38 | 55 | 7 | 4 | 1 | 780 |
| | 2004-2011 | 124 | 30 | 116 | 28 | 139 | 34 | 32 | 8 | 0 | 0 | 411 |
| 5 | 2012-2019 | 45 | 22 | 40 | 20 | 103 | 50 | 14 | 7 | 2 | 1 | 204 |
| | Total | 169 | 27 | 156 | 25 | 242 | 39 | 46 | 7 | 2 | 0 | 615 |
| | 2004-2011 | 108 | 33 | 77 | 24 | 118 | 36 | 22 | 7 | 1 | 0 | 326 |
| 6 | 2012-2019 | 39 | 26 | 25 | 17 | 71 | 48 | 11 | 7 | 2 | 1 | 148 |
| | Total | 147 | 31 | 102 | 22 | 189 | 40 | 33 | 7 | 3 | 1 | 474 |
| | 2004-2011 | 73 | 29 | 59 | 24 | 95 | 38 | 22 | 9 | 1 | 0 | 250 |
| 7 | 2012-2019 | 26 | 25 | 20 | 19 | 49 | 48 | 8 | 8 | 0 | 0 | 103 |
| | Total | 99 | 28 | 79 | 22 | 144 | 41 | 30 | 8 | 1 | 0 | 353 |
| | 2004-2011 | 56 | 28 | 46 | 23 | 74 | 37 | 20 | 10 | 3 | 2 | 199 |
| 8 | 2012-2019 | 19 | 26 | 16 | 22 | 31 | 42 | 7 | 10 | 0 | 0 | 73 |
| | Total | 75 | 28 | 62 | 23 | 105 | 39 | 27 | 10 | 3 | 1 | 272 |
| | 2004-2011 | 53 | 37 | 33 | 23 | 43 | 30 | 13 | 9 | 1 | 1 | 143 |
| 9 | 2012-2019 | 14 | 29 | 5 | 10 | 23 | 48 | 6 | 13 | 0 | 0 | 48 |
| | Total | 67 | 35 | 38 | 20 | 66 | 35 | 19 | 10 | 1 | 1 | 191 |
| | 2004-2011 | 42 | 37 | 25 | 22 | 35 | 31 | 11 | 10 | 0 | 0 | 113 |
| 10 | 2012-2019 | 9 | 30 | 7 | 23 | 11 | 37 | 3 | 10 | 0 | 0 | 30 |
| | Total | 51 | 36 | 32 | 22 | 46 | 32 | 14 | 10 | 0 | 0 | 143 |
| | 2004-2011 | 29 | 32 | 18 | 20 | 31 | 34 | 11 | 12 | 1 | 1 | 90 |
| 11 | 2012-2019 | 7 | 35 | 1 | 5 | 11 | 55 | 1 | 5 | 0 | 0 | 20 |
| | Total | 36 | 33 | 19 | 17 | 42 | 38 | 12 | 11 | 1 | 1 | 110 |
| | 2004-2011 | 14 | 21 | 15 | 22 | 29 | 43 | 10 | 15 | 0 | 0 | 68 |
| 12 | 2012-2019 | 7 | 50 | 1 | 7 | 5 | 36 | 1 | 7 | 0 | 0 | 14 |
| | Total | 21 | 26 | 16 | 20 | 34 | 41 | 11 | 13 | 0 | 0 | 82 |
| | 2004-2011 | 18 | 35 | 7 | 13 | 17 | 33 | 10 | 19 | 0 | 0 | 52 |
| 13 | 2012-2019 | 3 | 33 | 0 | 0 | 4 | 44 | 2 | 22 | 0 | 0 | 9 |
| | Total | 21 | 34 | 7 | 11 | 21 | 34 | 12 | 20 | 0 | 0 | 61 |
| | 2004-2011 | 14 | 31 | 6 | 13 | 17 | 38 | 8 | 18 | 0 | 0 | 45 |
| 14 | 2012-2019 | 2 | 33 | 0 | 0 | 4 | 67 | 0 | 0 | 0 | 0 | 6 |
| | Total | 16 | 31 | 6 | 12 | 21 | 41 | 8 | 16 | 0 | 0 | 51 |
| | 2004-2011 | 2 | 7 | 9 | 31 | 14 | 48 | 4 | 14 | 0 | 0 | 29 |
| 15 | 2012-2019 | 0 | 0 | 1 | 25 | 2 | 50 | 1 | 25 | 0 | 0 | 4 |
| | Total | 2 | 6 | 10 | 30 | 16 | 48 | 5 | 15 | 0 | 0 | 33 |

[a] First admission; [b] absolute frequency; [c] relative frequency (percentage).
