# Supplementary material for: A Descriptive Study of Repeated Hospitalizations and Survival of Patients with Metastatic Melanoma in the Northern Italian Region during 2004–2019
Source: Curr Oncol. 2023 May 25;30(6):5266–78. doi: 10.3390/curroncol30060400 (PMC10297154; doi:10.3390/curroncol30060400)
Supplement: Supplementary file 1 [file curroncol-30-00400-s001.zip › Melanoma Current Onc Table S3.pdf]

Table S3 – Type of hospitalization for patients with MM in Liguria Region during 2004-2019.

Readmission	Period	Planned		Urgent		IVC		Missing		Total
		N[b]	%[c]	N[b]	%[c]	N[b]	%[c]	N[b]	%[c]	
H_0 [a]	2004-2011	344	39	84	10	0	0	456	52	884
	2012-2019	307	45	89	13	0	0	290	42	686
	Total	651	41	173	11	0	0	746	48	1570
1	2004-2011	372	45	126	15	0	0	320	39	818
	2012-2019	303	50	95	16	0	0	205	34	603
	Total	675	48	221	16	0	0	525	37	1421
2	2004-2011	249	35	135	19	0	0	323	46	707
	2012-2019	144	31	95	20	0	0	231	49	470
	Total	393	33	230	20	0	0	554	47	1177
3	2004-2011	210	34	129	21	0	0	276	45	615
	2012-2019	80	21	105	28	1	0	188	50	374
	Total	290	29	234	24	1	0	464	47	989
4	2004-2011	143	28	134	26	0	0	233	46	510
	2012-2019	74	27	82	30	0	0	114	42	270
	Total	217	28	216	28	0	0	347	44	780
5	2004-2011	121	29	116	28	0	0	174	42	411
	2012-2019	39	19	64	31	0	0	101	50	204
	Total	160	26	180	29	0	0	275	45	615
6	2004-2011	97	30	91	28	0	0	138	42	326
	2012-2019	36	24	44	30	0	0	68	46	148
	Total	133	28	135	28	0	0	206	43	474
7	2004-2011	65	26	72	29	0	0	113	45	250
	2012-2019	30	29	36	35	0	0	37	36	103
	Total	95	27	108	31	0	0	150	42	353
8	2004-2011	66	33	45	23	0	0	88	44	199
	2012-2019	20	27	23	32	0	0	30	41	73
	Total	86	32	68	25	0	0	118	43	272
9	2004-2011	39	27	42	29	0	0	62	43	143
	2012-2019	12	25	19	40	0	0	17	35	48
	Total	51	27	61	32	0	0	79	41	191
10	2004-2011	26	23	36	32	0	0	51	45	113
	2012-2019	9	30	11	37	0	0	10	33	30
	Total	35	24	47	33	0	0	61	43	143
11	2004-2011	20	22	25	28	0	0	45	50	90
	2012-2019	3	15	6	30	0	0	11	55	20
	Total	23	21	31	28	0	0	56	51	110
12	2004-2011	22	32	13	19	0	0	33	49	68
	2012-2019	2	14	6	43	0	0	6	43	14
	Total	24	29	19	23	0	0	39	48	82
13	2004-2011	11	21	19	37	0	0	22	42	52
	2012-2019	4	44	2	22	0	0	3	33	9
	Total	15	25	21	34	0	0	25	41	61
14	2004-2011	13	29	9	20	0	0	23	51	45
	2012-2019	1	17	4	67	0	0	1	17	6
	Total	14	27	13	25	0	0	24	47	51
15	2004-2011	10	34	5	17	0	0	14	48	29
	2012-2019	0	0	1	25	0	0	3	75	4
	Total	10	30	6	18	0	0	17	52	33

[a] First admission; [b] absolute frequency; [c] relative frequency (percentage).
