# Supplementary material for: A Descriptive Study of Repeated Hospitalizations and Survival of Patients with Metastatic Melanoma in the Northern Italian Region during 2004–2019
Source: Curr Oncol. 2023 May 25;30(6):5266–78. doi: 10.3390/curroncol30060400 (PMC10297154; doi:10.3390/curroncol30060400)
Supplement: Supplementary file 1 [file curroncol-30-00400-s001.zip › Melanoma Current Onc Table S4.pdf]

Table S4. Reasons of day hospital admissions for patients with MM in Liguria Region during 2004-2019.

Readmission for day hospital	Period	Diagnostic		Surgery		Therapy		Other		Missing		Total
		N[b]	%[c]	N[b]	%[c]	N[b]	%[c]	N[b]	%[c]	N[b]	%[c]	
H_0[a]	2004-2011	63	13	215	46	130	28	38	8	26	6	472
	2012-2019	6	2	151	53	84	29	41	14	4	1	286
	Total	69	9	366	48	214	28	79	10	30	4	758
1	2004-2011	52	15	86	26	128	38	46	14	24	7	336
	2012-2019	4	2	35	18	143	72	17	9	0	0	199
	Total	56	10	121	23	271	51	63	12	24	4	535
2	2004-2011	52	16	68	20	158	48	25	8	29	9	332
	2012-2019	3	1	28	12	194	84	4	2	2	1	231
	Total	55	10	96	17	352	63	29	5	31	6	563
3	2004-2011	36	13	44	16	163	59	20	7	15	5	278
	2012-2019	4	2	31	17	145	78	4	2	1	1	185
	Total	40	9	75	16	308	67	24	5	16	3	463
4	2004-2011	31	13	37	16	149	63	7	3	13	5	237
	2012-2019	1	1	15	13	95	84	2	2	0	0	113
	Total	32	9	52	15	244	70	9	3	13	4	350
5	2004-2011	27	15	28	16	110	62	4	2	9	5	178
	2012-2019	1	1	8	8	88	89	2	2	0	0	99
	Total	28	10	36	13	198	71	6	2	9	3	277
6	2004-2011	18	13	17	12	89	62	7	5	12	8	143
	2012-2019	1	2	5	8	56	86	3	5	0	0	65
	Total	19	9	22	11	145	70	10	5	12	6	208
7	2004-2011	10	9	13	12	79	70	4	4	7	6	113
	2012-2019	1	3	1	3	34	92	1	3	0	0	37
	Total	11	7	14	9	113	75	5	3	7	5	150
8	2004-2011	9	10	11	12	59	66	3	3	8	9	90
	2012-2019	0	0	5	17	25	83	0	0	0	0	30
	Total	9	8	16	13	84	70	3	3	8	7	120
9	2004-2011	8	13	10	16	35	56	4	6	6	10	63
	2012-2019	0	0	1	6	16	94	0	0	0	0	17
	Total	8	10	11	14	51	64	4	5	6	8	80
10	2004-2011	6	11	9	17	31	58	1	2	6	11	53
	2012-2019	0	0	1	10	9	90	0	0	0	0	10
	Total	6	10	10	16	40	63	1	2	6	10	63
11	2004-2011	1	2	7	16	30	67	2	4	5	11	45
	2012-2019	0	0	0	0	11	100	0	0	0	0	11
	Total	1	2	7	13	41	73	2	4	5	9	56
12	2004-2011	2	6	7	21	23	70	0	0	1	3	33
	2012-2019	0	0	0	0	6	100	0	0	0	0	6
	Total	2	5	7	18	29	74	0	0	1	3	39
13	2004-2011	0	0	2	9	18	82	1	5	1	5	22
	2012-2019	0	0	0	0	3	100	0	0	0	0	3
	Total	0	0	2	8	21	84	1	4	1	4	25
14	2004-2011	3	13	3	13	16	70	0	0	1	4	23
	2012-2019	0	0	0	0	1	100	0	0	0	0	1
	Total	3	13	3	13	17	71	0	0	1	4	24
15	2004-2011	0	0	3	21	10	71	0	0	1	7	14
	2012-2019	0	0	0	0	3	100	0	0	0	0	3
	Total	0	0	3	18	13	76	0	0	1	6	17

[a] First admission; [b] absolute frequency; [c] relative frequency (percentage).
