# Supplementary material for: A Descriptive Study of Repeated Hospitalizations and Survival of Patients with Metastatic Melanoma in the Northern Italian Region during 2004–2019
Source: Curr Oncol. 2023 May 25;30(6):5266–78. doi: 10.3390/curroncol30060400 (PMC10297154; doi:10.3390/curroncol30060400)
Supplement: Supplementary file 1 [file curroncol-30-00400-s001.zip › Melanoma Current Onc Table S5.pdf]

Table S5. Chemotherapy in patients with MM in Liguria Region during 2004-2019.

Readmission	Period	No		Yes		Total
		N[b]	%[c]	N[b]	%[c]	
H_0[a]	2004-2011	776	88	108	12	884
	2012-2019	608	89	78	11	686
	Total	1384	88	186	12	1570
1	2004-2011	693	85	125	15	818
	2012-2019	460	76	143	24	603
	Total	1153	81	268	19	1421
2	2004-2011	539	76	168	24	707
	2012-2019	265	56	205	44	470
	Total	804	68	373	32	1177
3	2004-2011	440	72	175	28	615
	2012-2019	220	59	154	41	374
	Total	660	67	329	33	989
4	2004-2011	358	70	152	30	510
	2012-2019	172	64	98	36	270
	Total	530	68	250	32	780
5	2004-2011	285	69	126	31	411
	2012-2019	112	55	92	45	204
	Total	397	65	218	35	615
6	2004-2011	226	69	100	31	326
	2012-2019	90	61	58	39	148
	Total	316	67	158	33	474
7	2004-2011	163	65	87	35	250
	2012-2019	62	60	41	40	103
	Total	225	64	128	36	353
8	2004-2011	139	70	60	30	199
	2012-2019	45	62	28	38	73
	Total	184	68	88	32	272
9	2004-2011	102	71	41	29	143
	2012-2019	30	63	18	38	48
	Total	132	69	59	31	191
10	2004-2011	76	67	37	33	113
	2012-2019	21	70	9	30	30
	Total	97	68	46	32	143
11	2004-2011	60	67	30	33	90
	2012-2019	11	55	9	45	20
	Total	71	65	39	35	110
12	2004-2011	37	54	31	46	68
	2012-2019	9	64	5	36	14
	Total	46	56	36	44	82
13	2004-2011	34	65	18	35	52
	2012-2019	6	67	3	33	9
	Total	40	66	21	34	61
14	2004-2011	29	64	16	36	45
	2012-2019	5	83	1	17	6
	Total	34	67	17	33	51
15	2004-2011	15	52	14	48	29
	2012-2019	2	50	2	50	4
	Total	17	52	16	48	33

[a] First admission; [b] absolute frequency; [c] relative frequency (percentage).
